# A new laboratory surrogate (Monocyte Chemotactic Protein-1) for Disease Activity Score28: a favourable indicator for remission in rheumatoid arthritis

**DOI:** 10.1038/s41598-020-65127-5

**Published:** 2020-05-19

**Authors:** Lieh-bang Liou, Yao-Fan Fang, Chih Feng Tan, Jenn-Haung Lai, Shr-shian Jang, Ping-Han Tsai, Ting-chih Yeh

**Affiliations:** 1 Division of Rheumatology, Allergy and Immunology, Chang Gung Memorial Hospital at Linkou, Taoyuan, Taiwan; 2 Present Address: Chang Gung Memorial Hospital at Keelung, Keelung, Taiwan; 3 Department of Image Diagnostics and Therapeutics, Chang Gung Memorial Hospital at Linkou, Tao-yuan, Taiwan; 4 Chang Gung University College of Medicine, Tao-yuan, Taiwan

**Keywords:** Predictive markers, Rheumatoid arthritis

## Abstract

This prospective one-year follow-up study was conducted from 835 visits in 178 rheumatoid arthritis (RA) patients. Tender-/swollen-joint count, Health Assessment Questionnaire Disability Index (HAQ-DI), Disease Activity Score 28-ESR (DAS28-ESR), DAS28-CRP, Simplified Disease Activity Index (SDAI) and DAS28-monocyte chemotactic protein-1 (DAS28-MCP-1) scores were obtained every 3 months. Radiographs of hands and feet were acquired at baseline and one year. We evaluated the correlation and accuracy of activity scores in predicting remission, HAQ-DI changes and radiographic changes. DAS28-MCP-1 correlated strongly with DAS28-ESR, DAS28-CRP and SDAI scores (0.830, 0.899 and 0.931, respectively, with all *P* < 0.001). Score changes of DAS28-MCP-1 were comparable to those of DAS28-ESR, DAS28-CRP and SDAI in predicting changes in HAQ-DI and bone erosion. DAS28-MCP-1 (<2.2) was better than DAS28-ESR (<2.6) in indicating modified American Rheumatism Association remission and 2011 American College of Rheumatology/European League Against Rheumatism remission (75.61% vs. 36.99% and 81.71% vs. 49.13%, respectively) with odds ratios of 5.28 and 4.62 (both *P* < 0.001), respectively. We compared DAS28-MCP-1 with SDAI (≦3.3) in indicating remission with odds ratios of 2.63 (*P* = 0.002) and 0.98, respectively (and DAS28-MCP-1 with DAS28-CRP < 2.5: 1.33 and 0.92). Therefore, DAS28-MCP-1 is useful as an alternative in assessing RA activity.

## Introduction

Disease activity score (DAS) was simplified to DAS28 score^1^ after its initial implementation and validation in patients with rheumatoid arthritis (RA)^2,3^. The DAS28-ESR score, which includes erythrocyte sedimentation rate (ESR), has been used widely in clinical trials. DAS28-ESR scores are employed to define remission and non-remission, and low, moderate and high RA disease activity^4,5^. The European League Against Rheumatism (EULAR) response criteria for RA were developed from the DAS28-ESR score and are comparable in validity to the American College of Rheumatology (ACR) response criteria^6^. DAS28-ESR scores are now built into web-based and electronic patient-records, which simplify daily clinical practice^7^. The pathophysiological importance of C-reactive protein (CRP) and ESR differs. Therefore, CRP was proposed in an attempt to address weaknesses related to the use of ESR in the DAS28-ESR, such as immunoglobulin levels, fibrinogen levels, gender and anaemia, which could influence ESR values^8^. Later, studies indicated that optimal cut-off points for DAS28-ESR were higher than those for DAS28-CRP^9,10^.

Importantly, DAS28-ESR did not accurately discriminate remission from non-remission status^11^ and misclassified RA patients with moderate or high disease activity^12^. In particular, ESR and CRP values in DAS28 scores were within normal ranges in up to 40% of patients with RA^13^. To address these limitations, a number of other biomarkers have been studied in RA patients^14–17^. The modified DAS28-MCP-1 score (which includes monocyte chemotactic protein-1 [MCP-1] instead of ESR) was strongly correlated with DAS28-ESR and DAS28-CRP scores (0.984 and 0.971, respectively)^17^. In that report, we followed up a small group of RA patients further at Month 1, 3 and 6 (the patient number only at 44, 36 and 30, respectively: there were only a few remission visits available.): It revealed that DAS28-MCP-1 similarly correlated significantly with DAS28-ESR^17^. In particular, MCP-1 is produced locally at the inflammation site by activated monocytes and fibroblasts^18^, unlike ESR and CRP. Thus, we hypothesized that DAS28-MCP-1 might more accurately differentiate remission from non-remission for this study. This study also attempted to validate the DAS28-MCP-1 formula against the DAS28-ESR, DAS28-CRP and Simplified Disease Activity Index (SDAI) formulae, by longitudinal correlations (at Month 0, 3, 6, 9 and 12: hence, more remission visits could be included), by comparison through Bland–Altman plots and by means of Health Assessment Questionnaire–Disability Index (HAQ-DI) and radiographic changes.

## Results

### Baseline characteristics of the patients

Our RA patients’ demographic and clinical characteristics were displayed (Table 1). The RA patients were treated with standard disease-modifying anti-rheumatic drugs, namely sulfasalazine, methotrexate and hydroxychloroquine. Biologics (mainly etanercept, adalimumab and golimumab; only two patients received abatacept) were used in 27.0% of RA patients.

**Table 1:** Baseline characteristics of enrolled rheumatoid arthritis patients.

Variables	Mean ± S.D. or median with inter-quartile ranges	Range
Number of patients	178	F:M = 3.94:1 (142:36)
Total visits in 12 months	835	
Age (year-old)	54 (46, 61)	20–80
Disease Duration^a^ (months)	81.00 (20.25, 164.25)	2.00–415.00
Tender joint count	5.0 (2.0, 9.3)	0.0–28.0
Swollen joint count	2.0 (1.0, 5.3)	0.0–24.0
Global health	50 (20, 60)	0–100
ESR (mm/hr)	17.50 (9.75, 36.00)	2.00–100.00
MCP-1 (pg/mL)	101.8 (73.6, 140.1)	18.7–2074.4
Hs-CRP (mg/L)	3.90 (1.13, 9.21)	0.24–133.15
Rheumatoid factor (IU/mL, positive rate)	22.5 (10.28, 136.00) (43.8%)	9.88–1200.00
IgG anti-CCP (mg/dL, positive rate)	194.8 (5.5, 277.2) (31.5%)	1.9–327.9
DAS28-ESR scores	4.3 ± 1.4	0.8–7.3
DAS28-MCP-1 scores	4.2 ± 1.0	2.2–6.8
DAS28-CRP scores	4.8 ± 1.3	2.5–8.3
SDAI	15.3 (9.1, 23.7)	0.2–68.1
HAQ-DI	0.167 (0.000, 0.359)^b^	0.00–1.85
Prednisolone dose (mg/day, %^c^)	2.50 (1.88, 5.00) (42.1%)	1.25–20.00
Sulfasalazine (mg/day, %^c^)	2500 (2000, 3000) (73.6%)	500–3000
Methotrexate (mg/week, %^c^)	12.5 (7.5, 15.0) (68.0%)	5.0–20.0
Hydroxychloroquine (mg/day, %^c^)	400.0 (237.5, 400.0) (37.1%)	200.0–500.0
Biologics^d^	27.0%	

^a^Definition: see Methods; DAS28-ESR: Disease Activity Score28 with inclusion of ESR, DAS28-MCP-1: score including MCP-1, DAS28-CRP: score including CRP, SDAI: Simplified Disease Activity Index, HAQ-DI: health assessment questionnaire-disability index, all recorded at time of enrollment. Normal ranges: ESR < 15 mm/hr, MCP-1 (0.0–103.7 pg/mL, ref. ^17^), hs-CRP level <5 mg/L, rheumatoid factor <15 IU/mL, IgG anti-CCP < 20 mg/dL. ^b^The 10^th^ and 90^th^ percentiles were at 0.000 and 0.710. ^c^Indicates % of RA patients using that medication at enrollment (for 3 months or longer). ^d^Mainly etanercept, adalimumab and golimumab.

The rate of participation during the 12-month follow-up period was 100.0% (178/178) at baseline, 91.6% (163/178) at three months, 91.6% (163/178) at six months, 91.0% (162/178) at nine months and 94.9% (169/178) at twelve months. The patients missed scheduled outpatient visits for personal reasons; hence, it occurred randomly. Radiographs of the hands and feet were obtained from 100.0% (178/178) of RA patients at baseline and 94.9% (169/178) of patients at twelve months.

### Correlation of DAS28-MCP-1 scores with DAS28-ESR, DAS28-CRP and SDAI scores

**Figure 1:**
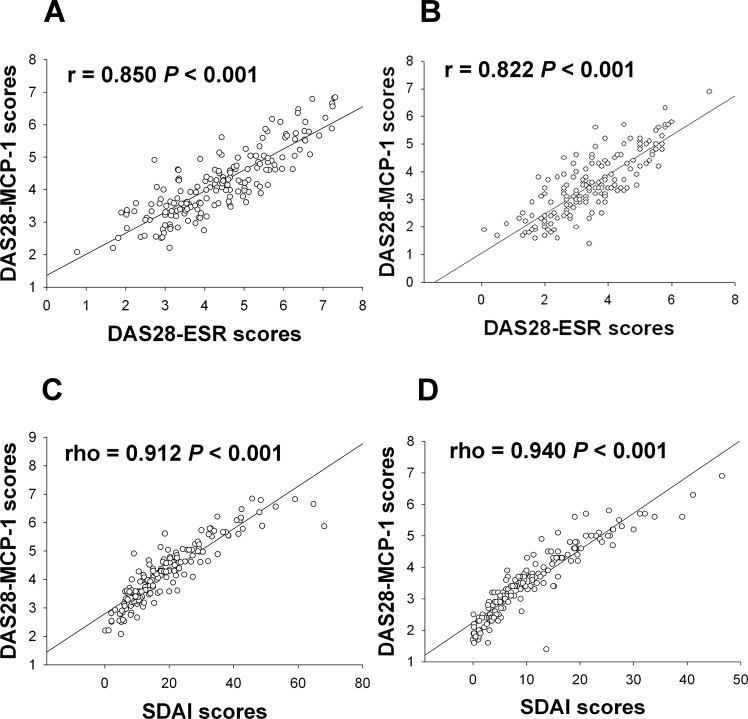
Correlation between DAS28-MCP-1 and DAS28-ESR scores or and Simplified Disease Activity Index (SDAI) scores during the study period. The correlation between DAS28-MCP-1 and DAS28-ESR scores is displayed at (**A)** Month 0 (baseline) (n = 178) and (**B)** Month 12 (n = 169). The correlation between DAS28-MCP-1 and SDAI scores is presented at (**C)** Month 0 (baseline) (n = 178) and (**D)** Month 12 (n = 169).

DAS28-MCP-1 positively correlated with DAS28-ESR, DAS28-CRP and SDAI for 835 visits and at all time points (Fig. 1, Table 2 and eTable 1 in the Supplement) (formulae as in refs. ^3,19^). The baseline correlation coefficient between DAS28-MCP-1 and DAS28-ESR or DAS28-CRP was slightly lower than that reported in a previous cross-sectional study^17^. At all time points, the strength of association between DAS28-MCP-1 scores and DAS28-ESR scores was higher for women than men and higher for rheumatoid factor (RF)-positive patients than RF-negative patients, though the association were not significant except for one (eTable 2 in the Supplement)^20,21^.

**Table 2:** Correlation of DAS28-MCP-1 scores with DAS28-ESR and DAS28-CRP scores with different ESR/CRP cut-off points.

DAS28-MCP-1	Time points	DAS28-ESR	DAS28-ESR (ESR ≧28 mm/hr)	DAS28-ESR (ESR <28 mm/hr)	DAS28-CRP	DAS28-CRP (CRP ≧10 mg/L)	DAS28-CRP (CRP <10 mg/L)
	All 835 visits	0.830^a^ (0.808–0.850)	0.949^b^ (0.934–0.961)	0.865^b^ (0.843–0.884)	0.899^a^ (0.885–0.911)	0.964^c^ (0.951–0.975)	0.920^c^ (0.907–0.931)
correlates with	Baseline	0.850	0.940	0.874	0.901	0.945	0.926
other scores at	Month 3	0.840	0.962	0.856	0.901	0.949	0.922
different months	Month 6	0.862	0.964	0.876	0.910	0.962	0.933
	Month 9	0.818	0.897	0.841	0.893	0.969	0.898
	Month12	0.822	0.939	0.849	0.887	0.976	0.902
Biologics^d^	All 232 visits	0.779^d^ (0.723–0.825)	0.947^e^ (0.920–0.965)	0.806^e^ (0.740–0.857)	0.892^d^ (0.875–0.923)	0.919^f^ (0.875–0.935)	0.860^f^ (0.837–0.880)

All values are Pearson correlation coefficients, except where indicated. ^a^Spearman’s correlation coefficients; the 95% confidence interval (CI) in parentheses: *P* < 0.001 (and all below were done as in ref. ^20,21^). ^b^The 95% CI: *P* < 0.001. ^c^The 95% CI: *P* < 0.001. ^d^Visits receiving biologics; the 95% CI: *P* < 0.001. ^e^The 95% CI: *P* < 0.001. ^f^The 95% CI: *P* = 0.042.

### Criterion validity

#### Agreement in stratified analysis

When RA patients were stratified by ESR values (ESR ≧ 28 mm/hr vs. ESR < 28 mm/hr, indicating high vs. low ESR, respectively, as described in Methods)^22^, the correlation coefficient between DAS28-MCP-1 score and DAS28-ESR score increased to 0.949 for 835 visits and to 0.940 at baseline (Table 2). The trend was similar for other time points and for patients receiving biologics (Table 2). Likewise, the trend was seen for the correlation of DAS28-MCP-1 score with DAS28-CRP score, especially for patients with a CRP concentration of **≧**10 mg/L (Table 2).

#### Limits of agreement

**Figure 2:**
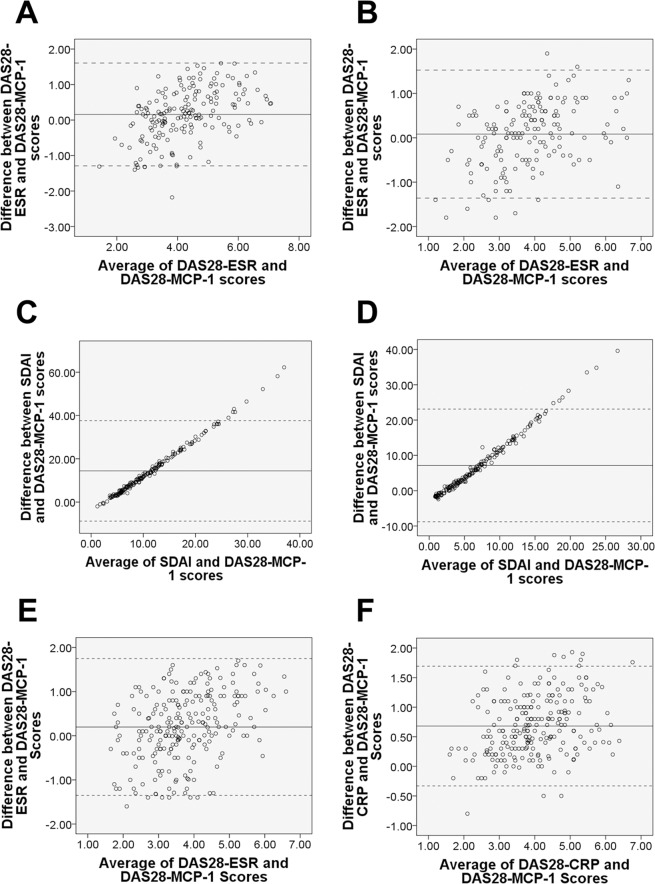
Bland–Altman plots of DAS28-ESR, SDAI and DAS28-CRP vs. DAS28-MCP-1 scores. The solid central line represents the mean difference between two scores. The upper and lower dotted lines indicate 95% limits of agreement (2 SD from the mean difference). (**A)** Baseline of DAS28-ESR vs. of DAS28-MCP-1 scores: below 2 SD = 2.2%, above 2 SD = 0.0%; (**B)** Month 12 of DAS28-ESR vs. of DAS28-MCP-1 scores: below 2 SD = 3.6%, above 2 SD = 0.6%. **(C)** Baseline of SDAI vs. of DAS28-MCP-1 scores: below 2 SD = 0.0%, above 2 SD = 4.5%; (**D)** Month 12 of SDAI vs. of DAS28-MCP-1 scores: below 2 SD = 0.0%, above 2 SD = 4.1%. (**E)** The biologics subgroup of DAS28-ESR vs. of DAS28-MCP-1 scores: below 2 SD = 2.6%, above 2 SD = 0.0%; (**F)** The biologics subgroup of DAS28-CRP vs. of DAS28-MCP-1 scores: below 2 SD = 1.3%, above 2 SD = 3.9%.

**Figure 3:**
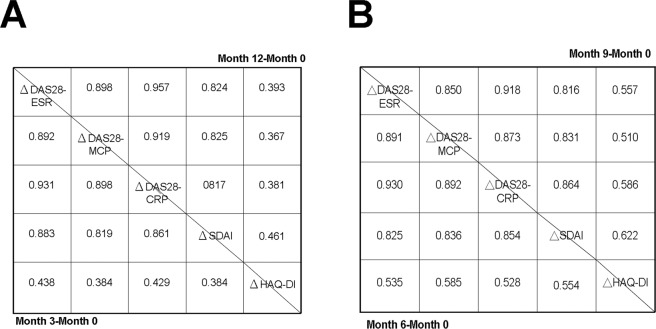
Correlation of changes in different kinds of DAS28 scores/SDAI with changes in the Health Assessment Questionnaire–Disability Index (HAQ-DI). △DAS28-ESR: change in DAS28 score with inclusion of ESR; △DAS28-MCP: change in DAS28-MCP-1 score; △DAS28-CRP: change in DAS28-CRP score; △SDAI: change in Simplified Disease Activity Index; △HAQ-DI: change in HAQ-DI. Correlation coefficients below the diagonal line are for (**A**) Month 3 minus Month 0 (Month 3-Month 0; the latter is Baseline) and (**B**) Month 6 minus Month 0 (Month 6-Month 0). Correlation coefficients above the diagonal line are for (**A**) Month 12 minus Month 0 (Month 12-Month 0) and (**B**) Month 9 minus Month 0 (Month 9-Month 0). Correlations with SDAI and HAQ-DI were analysed by Spearman correlation; other correlations were analysed by Pearson correlation analysis. All *P*-values were less than 0.001.

In Bland–Altman plots, the mean difference in measurements of DAS28-ESR and DAS28-MCP-1 ranged from −0.07 to 0.16 at all time points (Fig. 2A,B). This indicates strong agreement between the two DAS28 formulae. The percentage of measurements beyond the 95% limits of agreement for DAS28-ESR and DAS28-MCP-1 scores ranged from 2.2% (at baseline) to 5.5% (Month three). Compared to DAS28-MCP-1 scores, low DAS28-ESR scores (defined as scores two or more SD below the mean difference) were more common than high DAS28-ESR scores (Fig. 2A,B). Similarly, low discordance beyond the 95% limits of agreement was found for the biologics subgroup (Fig. 2E,F: the latter showing a high agreement between DAS28-CRP and DAS28-MCP-1). Only higher SDAI scores than DAS28-MCP-1 scores were found with an explicit diagonal line in Bland–Altman plots; due to the SDAI scores being about three times higher of the DAS28-MCP-1 scores (Fig. 2C,D). All the above findings indicate that DAS28-MCP-1 scores are highly congruent with DAS28-ESR and SDAI scores (Table 2, eTables 1–3 in the Supplement and Figs. 1–3), and with DAS28-CRP scores (Table 2, eTables 1 and 3 in the Supplement, Figs. 2F and 3).

#### True remission rates of different kinds of DAS28 measurements

DAS28-MCP-1 score-based remission was selected at <2.2 to avoid over-treatment, based on the 2011 American College of Rheumatology/European League Against Rheumatism (ACR/EULAR) definition of non-remission setting at ≦5.00% (eTable 4 in the Supplement), to match the comparable 2011 ACR/EULAR remission^23^ (see Methods) rates as those of DAS28-CRP and SDAI (Table 3) and to fit the DAS28-MCP-1 < 2.2 fulfilling ≧90.0% of SDAI remission by the 2011 ACR/EULAR definition (Table 4). For patients who satisfied the five criteria for modified American Rheumatism Association (ARA) remission at each visit (Methods and ref. ^5^), DAS28-MCP-1 scores of <2.2 (remission status) rendered the highest rate of modified ARA remission and scores of ≧2.2 (non-remission status) provided a low rate of modified ARA remission (Table 3). The true modified ARA remission rate of DAS28-MCP-1 scores <2.2 was significantly higher than those of DAS28-ESR-based and SDAI-based remission, but it was similar to DAS28-CRP–based remission (Table 3). Surprisingly, only one third of visits for DAS28-ESR-defined remission status fulfilled the modified ARA criteria (2.6, as described)^4^. The 2011 ACR/EULAR definition of remission yielded similar results: a DAS28-MCP-1 score <2.2 yielded one of the highest remission rates; it was similar to those of DAS28-CRP–based remission and SDAI-based remission (Table 3).

**Table 3:** Remission rates by different criteria in different Disease Activity Score28 (DAS28) score-based statuses.

	Remission status	Odds Ratio^a^	Non-remission status
**Fulfillment of modified ARA remission**^**b**^			
DAS28-ESR (<2.6)	36.99% (64/173)	Reference	0.45% (3/662)
DAS28-MCP-1 (<2.2)	75.61% (62/82)	5.28	0.80% (6/753)
DAS28-CRP (<2.5)	70.00% (49/70)	3.97	2.35% (18/765)
SDAI (≦3.3)	54.10% (66/122)	2.01	0.14% (1/713)
**Fulfillment of 2011 ACR/EULAR remission definition**^**c**^			
DAS28-ESR (<2.6)	49.13% (85/173)	Reference	2.27% (15/662)
DAS28-MCP-1 (<2.2)	81.71% (67/82)	4.62	3.98% (30/753)
DAS28-CRP (<2.5)	82.86% (58/70)	5.00	5.49% (42/765)
SDAI (≦3.3)	81.97% (100/122)	4.71	0.00% (0/713)

The remission status (corresponds to positive predictive values in Tables 5 and 6) of patients with rheumatoid arthritis was classified by indicated cut-offs and percentages calculated in each category. Abbreviations were the same as in Table 1. ^a^All odds ratios vs. the reference in each category gave *P* < 0.005. ^b^Satisfied the modified American Rheumatism Association (ARA) remission criteria during 835 visits (ref. ^5^). Inside parentheses are visit numbers. DAS28-MCP-1 vs. SDAI: *P* = 0.002 (95% C.I.: 1.42–4.88); DAS28-MCP-1 vs. DAS28-CRP: *P* = 0.438 (95% C.I.: 0.65–2.72). ^c^American College of Rheumatology/European League against Rheumatism (ACR/EULAR): as in ref. ^23^. DAS28-MCP-1 vs. DAS28-CRP and SDAI rendered both *P* > 0.500.

**Table 4:** Percentages of different DAS28-ESR and DAS28-MCP-1 cut-points based remission fulfilled SDAI-defined remission.

Category cut-points	Percentages fulfilled SDAI score ≦ 3.3^a^
DAS28-ESR < 2.6	56.1% (97/173)
DAS28-ESR < 2.4	60.1% (86/143)
DAS28-ESR < 2.2	67.3% (76/113)
DAS28-ESR < 2.0	72.1% (62/86)
DAS28-MCP-1 < 2.6	74.3% (104/140)
DAS28-MCP-1 < 2.4	86.0% (86/100)
DAS28-MCP-1 < 2.2	93.9% (77/82)
DAS28-MCP-1 < 2.0	95.0% (57/60)

Abbreviations were the same as in Table 1. ^a^As defined in American College of Rheumatology/European League against Rheumatism remission definition (ref. ^23^). Numbers in parentheses indicate fulfilment visits divided by total visits in each category.

### Construct validity

#### Correlation of different kinds of DAS28 scores and SDAI with HAQ-DI

DAS28-MCP-1 significantly correlated with HAQ-DI. Similarly, DAS28-ESR, DAS28-CRP and SDAI correlated with HAQ-DI. Correlations were not significantly different (eTable 1 in the Supplement). In the biologics subgroup, DAS28-ESR, DAS28-MCP-1, DAS28-CRP and SDAI correlated with HAQ-DI at rho = 0.440, 0.563, 0.592, and 0.624, respectively.

Furthermore, our analysis revealed a similar trend of correlation between change in different kinds of DAS28 scores/SDAI and change in HAQ-DI (Fig. 3). These findings indicate that the patterns of correlation between different kinds of DAS28 scores/SDAI and HAQ-DI were quite similar.

#### Area-under-the-curve (AUC) of different kinds of DAS28 score/SDAI changes against radiographic changes

Whether the differences in DAS28-ESR, DAS28-MCP-1, DAS28-CRP, SDAI and HAQ-DI scores between individual Month three, six, nine and twelve and Month 0 mirrored changes in bone erosion between Month 12 and Month 0 was further investigated by receiver-operating-characteristics (ROC) (eTable 5 in the Supplement). All AUCs were from 0.422 to 0.557 and the diagnostic accuracy was either bad or not useful (eTable 5 in the Supplement). That is, the ROC performance of different kinds of DAS28/SDAI changes against radiographic changes was not different. Similarly, all DAS/SDAI and HAQ-DI scores against modified total Sharp score (mTSS, with the cut-off at 6, which was greater than the smallest detectable difference [SDD] at 5.86 for mTSS, as in Methods) rendered all AUCs smaller than 0.5.

#### The performance of the different scores to detect remission according to two remission definitions

The sensitivity, specificity, positive predictive value (PPV), negative predictive value (NPV), Youden index, diagnostic effectiveness and diagnostic odds ratio were calculated based on the modified ARA remission definition^5^ (Table 5) and the 2011 ACR/EULAR remission definition^23^ (see Methods) (Table 6). In terms of global measures of diagnostic accuracy (Youden index, diagnostic effectiveness and diagnostic odds ratio), the performance of SDAI appeared to be the best (Tables 5 and 6). DAS28-MCP-1 did perform well, close or second to SDAI. We also show positive predictive values (represented as remission status %) in Table 3.

**Table 5:** The performance of different activity score-based remissions fulfilling modified ARA remission definition. PPV: positive predictive value; NPV: negative predictive value.

	DAS28-ESR	DAS28-MCP-1	DAS28-CRP	SDAI
Sensitivity	95.52%	91.18%	73.13%	98.51%
Specificity	85.81%	97.39%	97.27%	92.71%
PPV	36.99%	75.61%	70.00%	54.10%
NPV	99.55%	99.20%	97.65%	99.86%
Youden index	0.81	0.89	0.70	0.91
Diagnostic effectiveness	0.87	0.97	0.94	0.93
Diagnostic odds ratio	128.98	385.95	96.83	839.14

**Table 6:** The performance of different activity score-based remissions fulfilling 2011 ACR/EULAR remission definition.

	DAS28-ESR	DAS28-MCP-1	DAS28-CRP	SDAI
Sensitivity	85.00%	69.07%	58.00%	100%
Specificity	88.03%	97.97%	98.37%	97.01%
PPV	49.13%	81.71%	82.86%	81.97%
NPV	97.73%	96.02%	94.51%	100%
Youden index	0.73	0.67	0.56	0.97
Diagnostic effectiveness	0.88	0.95	0.94	0.97
Diagnostic odds ratio	41.66	107.65	83.20	infinite

PPV: positive predictive value; NPV: negative predictive value.

## Discussion

Acute-phase reactants like ESR and CRP can be affected by a complicated array of factors and are produced far from the site of inflammation. However, MCP-1 is produced by activated monocytes and fibroblasts at the site of inflammation^18^. Furthermore, an MCP-1 antagonist suppressed or prevented inflammatory arthritis in MRL/lpr mice^24^, which suggests that MCP-1 is important in arthritic inflammation. In RA patients with high laboratory values (ESR ≧28 mm/hr or CRP ≧ 10 mg/L), DAS28-MCP-1 score correlated strongly with DAS28-ESR and DAS28-CRP scores (Table 2), which is consistent with the very high correlation coefficients (0.984 and 0.971. respectively) reported in a previous study^17^. These findings were further supported by the similar sensitivity in change of these three DAS28 scores and SDAI to functional and radiographic changes (Fig. 3 and eTable 5 in the supplement). Interestingly, DAS28-MCP-1 was more strongly correlated with DAS28-CRP than with DAS28-ESR and their correlation coefficients significantly different (Table 2), probably because local tissue production of MCP-1 and cytokines (which induce CRP secretion) is similar^8,18^.

The association between DAS28-MCP-1 and DAS28-ESR was stronger but not significant in women than men (eTable 2 in the Supplement), which suggests sex differences in the characteristics of RA^25^. It is unclear why high ESR, high CRP and positive RF (Table 2 and eTable 2 in the Supplement) are associated with a stronger correlation between DAS28-MCP-1 and DAS28-ESR or DAS28-CRP. Similar to previous reports^5,11,26^, DAS28-ESR was unable to distinguish remission from non-remission (Table 3). Intriguingly, DAS28-MCP-1 was superior to DAS28-ESR and SDAI in identifying modified ARA remission and better than DAS28-ESR in indicating 2011 ACR/EULAR remission definition (Table 3).

Moreover, the precision of remission definition employing new lower cut-points from two recent reports^26,27^ was analysed in this study. The result of using DAS28-CRP cut-point at 1.9 was 100.00% (20/20 vistis) for modified ARA remission definition and 100.00% (20/20 visits) for 2011 ACR/EULAR remission definition. Using DAS28-ESR cut-point at 2.2, the proportion that met modified ARA remission definition was 50.44% and that fulfilled 2011 ACR/EULAR remission definition was 61.95% (eTable 4 in the Supplement). The true remission rates with different DAS28-ESR cut-points were all lower than those given by DAS28-MCP-1 with the same cut-points (eTable 4 in the Supplement). It indicates that DAS28-ESR with the same cut-points for remission status does not match those of DAS28-MCP-1 by the two remission definitions. Hence, the results of our analysis using new lower cut-points for DAS28-CRP-remission differed from those of Schoels M *et al*.^26^ and of Fleischmann R *et al*.^27^. The latter two studies were clinical trials, unlike our study. The DAS28-CRP remission cut-point at 1.9 rendered only 20 visits out of 835 visits met for remission definitions, which may limit the benefits for clinical practice.

Using PASS Software, the statistical power was 1.00 for the correlation of DAS28-MCP-1 and DAS28-ESR at baseline (n = 178) and of DAS28-MCP-1 and DAS28-CRP at baseline (n = 178) (Table 2). Moreover, the power was also 1.00 for DAS28-MCP-1’s correlation with DAS28-ESR at baseline for women and men (eTable 2 in the Supplement). Additionally, the correlation of DAS28-MCP-1 and DAS28-ESR at baseline for RF-positive patients and RF-negative patients resulted in a power of 1.00 (eTable 2 in the Supplement). Therefore, our sample size is sufficient to perform the analyses.

In the functional assessment, DAS28-ESR, DAS28-MCP-1, DAS28-CRP and SDAI scores in women were similar in differentiating meaningful radiographic progression damage-relevant HAQ-DI changes from non-progression HAQ-DI changes (HAQ-DI cut at 1.44, ref. ^28^) across several time points (eTable 3 in the Supplement). No meaningful AUCs were seen in men. These results also suggest a gender difference in RA disease activity^25^. Nevertheless, it is known that the HAQ-DI score does not discriminate between function associated with inflammation and function associated with damage. AUCs for changes in DAS28-ESR, DAS28-MCP-1 and DAS28-CRP between different months against change in bone erosion (eTable 5 in the supplement) were similar to those reported^1^. Moreover, in doing aliquots of one year for disease duration, the highest correlation coefficient between different DAS28 scores/SDAI and HAQ-DI scores was not found in RA patients with disease duration less than one year (eTable 6 in the Supplement), as described^29^.

The strengths of the modified DAS28-MCP-1 formula include: more accurate identification of true remission compared with DAS28-ESR by two kinds of remission definition (Table 3). Second, this is the first report demonstrating that DAS28-MCP-1 correlated (also with different time-point changes) with DAS28-ESR, DAS28-CRP and SDAI at five times points in a year. Third, the 178 RA patients recruited at baseline followed a normal distribution based on classification of disease activity score (see Statistical analyses), commonly seen in daily clinic. This study is limited by its small sample size for remission visits although these limitations are partly offset by the large number of assessments (835 visits in total), which were used to define true remission rates (Table 3). Additionally, patients enrolled at Month three, six, nine and twelve had similar mean age and disease duration (the definition: see Methods) to missing patients (eTable 7 in the Supplement). Hence, we considered the potential bias influenced by age and disease duration to be minimal. Last, the correlation of DAS28-MCP-1 with other clinical combined scores was not limited to those using the same DAS28 formula with partial modification, but also extended to SDAI scores using a different formula. In particular, our patients were from different regions of Taiwan, which may represent the general population of Taiwan. Nevertheless, a future study with a large sample size for remission visits is warranted to confirm the current findings. In particular, the validation of DAS28-MCP-1 against DAS28-ESR, DAS28-CRP and SDAI in monitoring the responses of medications, especially biologics (above all, Tocilizumab users)^26^, in rheumatoid arthritis is much needed.

We further analysed different DAS cuff-points based remission in Table 3 against 2011 ACR/EULAR non-remission. It showed that SDAI-based remission (≦3.3) against 2011 ACR/EULAR non-remission gave an AUC of 0.875 (95% C.I.: 0.807–0.942); DAS28-CRP-based remission (<2.5) provided an AUC of 0.825 (95% C.I.: 0.709–0.940); DAS28-MCP-1-based remission (<2.2) offered an AUC of 0.543 (95% C.I.: 0.369–0.717). That is, SDAI-based remission and DAS28-CRP-based remission could further discriminate 2011 ACR/EULAR non-remission within their own groups, in contrast to DAS28-MCP-1-based remission. Hence, SDAI-based remission and DAS28-CRP-based remission have a potential to be sub-divided into sub-groups; may be volatile to change (hence, unstable) of their remission cut-off points to better fulfill 2011 ACR/EULAR remission in the future. However, DAS28-MCP-1-based remission is less likely to be subdivided into 2011 ACR/EULAR non-remission and remission. That is, DAS28-MCP-1-based remission is more stable in terms of fulfillment of 2011 ACR/EULAR remission than SDAI-based remission and DAS28-CRP-based remission though these three had similar 2011 ACR/EULAR remission rates (Table 3). Whether DAS28-MCP-1 performs similarly to DAS28-CRP and SDAI in monitoring medication responses needs further studies.

A major concern of using DAS28-MCP-1 in clinical practice may be the higher cost of MCP-1 assay compared to CRP test. The cost of MCP-1 test by ELISA assay is 0.58 times lower than the cost of CRP examination by turbimetry (which is the current method used in our Hospital system’s four hospital clusters [nine separate branches] across Taiwan). However, the cost of MCP-1 assay is the same as the cost of CRP examination by ELISA assay. Another consideration is that a rapid and high-throughput laboratory machine for the examination of MCP-1 is still unavailable. Nevertheless, an increase in the demand for MCP-1 examination method may lead to an improvement in the laboratory technology.

The modified DAS28-MCP-1 formula has obvious correlation validity, functional validity, radiographic validity and comparable sensitivity to HAQ-DI and radiologic changes with other DAS28 formulae and SDAI. This is the first longitudinal study that compares a modified DAS28 formula, which incorporates an inflammatory biomarker (MCP-1) with other presently used DAS28 formulae and SDAI during a 12-month period. This study enrolled adult RA patients with a few exclusions (as described in Methods), unlike those in clinical trials, and its results may be applied to daily clinical practice.

In summary, our findings suggest that the modified DAS28-MCP-1 formula can be used in the evaluation of RA disease activity. Moreover, DAS28-MCP-1 score should be helpful for rheumatologists worldwide to confidently identify true remission of RA and efficiently monitor therapeutic responses in the future, although more studies are needed for confirmation in different racial and ethnic groups.

## Methods

### Study design

This is a different RA patient cohort from the cohort in the original study published in 2013^17^. The study was conducted at the Linkou and Taipei branches of Chang Gung Memorial Hospital, Taiwan, at a medical centre level, during the period from July 2013 through December 2016. The institutional review board of Chang Gung Memorial Hospital approved the study protocol with adherence to an appropriate version of the 1964 Helsinki Declaration. All methods were executed in accordance with the relevant guidelines and regulations. RA patients who fulfilled the 1987 ACR criteria for RA and had at least one tender joint or one swollen joint with a greater potential for DAS28-ESR score changes were selected as candidates after they provided written informed consent. Patients with no tender or swollen joint and younger than 20 or older than 80 years old were excluded. RA patients between 20 and 80 years old were randomly enrolled (consecutive and volunteered) from our adult rheumatology outpatient department with a few exclusions (see above). They underwent follow-up assessments, including blood collection, every 3 months for a period of 12 months (5 visits per patient). There were in total 835 visits in 178 RA patients with medication given at individual rheumatologist’s discretion (Table 1).

At each visit, clinicians collected data on current medications (first visit only), HAQ-DI items, morning stiffness, tender-joint count (TJC), swollen-joint count (SJC), patient’s and evaluator’s global assessment of disease activity (visual-analogue scale [VAS; in cm] as PGA [global health in Table 1] and EGA), ESR, CRP and MCP-1. Radiographic examination of hands and feet was done at baseline and one year. Radiographs were collected with a Toshiba Digital Radiography system (KXO-50R, 2003) and recorded with a GE Picture Archiving and Communication System (GE RA1000, 2003). Pincus T *et al*. had defined severe inflammation as a blood CRP level ≧10 mg/L or an ESR ≧ 28 mm/hr^22^. A CRP level <10 mg/L or an ESR < 28 mm/hr was considered mild inflammation. However, to avoid dispute, we refer to these two categories as high or low CRP and ESR. Moreover, our Hospital laboratory has adopted ARA remission criteria for people after age 50: men <20 mm/hr and women <30 mm/hr as normal^4^. Some RA patients did not come back at scheduled outpatient visits as potential bias (see Statistical analyses and Discussion).

### Validity of HAQ-DI

The previously described Chinese-language version of the HAQ-DI was used in the clinical assessment^30,31^.

### Measurement of serum MCP-1, RF and IgG anti-CCP levels

Serum MCP-1 from RA patients was examined in duplicate by ELISA assay (mean values taken) in accordance with the manufacturer’s recommendations (R&D systems, Minneapolis, MN, USA). At the first visit, RF (measured by nephelometry with N Latex RF Kit from Siemens Healthcare Diagnostics Products GmbH, Marburg, Germany) and anti–cyclic citrullinated peptide (anti-CCP) antibodies (Quanta Lite CCP3 IgG ELISA kit; Inova Diagnostics, Inc., San Diego, CA, USA) were examined. Normal ranges: MCP-1 (0.0–103.7 pg/mL: mean plus two standard deviations, ref. ^17^).

### Calculation of different DAS28 and SDAI scores

DAS28-ESR scores (= [0.56 × √TJC] + [0.28 × √SJC] + 0.70 × ln[ESR] + 0.014 × PGA [in mm]), DAS28-CRP scores (= [0.56 ×√TJC] + [0.28 ×√SJC] + (0.36 × ln[CRP; in mg/l]) + 1) + (0.014 × PGA [in mm]) + 0.96) and SDAI ( = SJC + TJC + PGA [VAS; in cm] + EGA [VAS; in cm] + CRP [in mg/dL]) were calculated as previously described^3,19^. DAS28-MCP-1 score was calculated with the following modified DAS28 formula: DAS28-MCP-1 = 0.56 × √TJC + 0.28 × √SJC + 0.39 × ln(MCP-1; in pg/mL) + 0.014 × (PGA [in mm])^17^.

### Determination of remission rates by the DAS28-MCP-1 formula and other DAS28/SDAI formulae

Previously described modified ARA remission criteria were used^5^, namely, morning stiffness of ≦15 minutes, pain scoring by visual analogue scale of ≦10 mm, 28-tender-joint count = 0, 28-swollen-joint count = 0, and ESR < 30 for women or <20 mm for men. When these five criteria were satisfied with no requirement of two or three-month follow-up^5^, DAS28-MCP-1 scores of 2.2 (see Results: True remission rates of different kinds of DAS28 measurements and Discussion) were used to classify remission status. In addition, remission was also defined by using the 2011 Boolean ACR/EULAR definition of remission with all following score measures ≦1: TJC, SJC, CRP (in mg/dL), and PGA (0–10 scale)^23^.

### Radiographic assessment

Radiographs of hands and feet were obtained at baseline and 12-month and assessed by two blinded evaluators. Presence of bone erosion was recorded without knowledge of patient clinical condition and the mean of the two evaluators’ scores was recorded, according to van der Heijde’s previously described modification of Sharp’s method^32^. The interclass correlation coefficient for measurement of mTSS on the combined 12-month and baseline radiographs was 0.96. Systematic error was 0.88 units and the SDD in the measurement error (95% level of agreement) was 5.86 units. Moreover, the interclass correlation coefficient for measurements of bone erosion on the combined 12-month and baseline radiographs was 0.93. Systematic error was 0.44 units and the SDD in the measurement error (95% level of agreement) was 2.90 units.

### Statistical analyses

When 178 RA patients at baseline were classified by DAS28-ESR as 3 major subgroups: remission plus low disease activity (<2.6 and between ≧2.6 and <3.2: together 21.91% = 39/178), moderate disease activity (between ≧3.2 and <5.1: 49.44% = 88/178) and high disease activity (≧5.1: 28.65% = 51/178): it followed a near-normal distribution with peak at the moderate disease activity subgroup. This was supported by the one-sample Kolmogorov–Smirnov Z test of baseline DAS28-ESR scores at 0.813 and asymptotic significance at 0.524, indicating of normal distribution. Hence, this reflected an appropriate RA patient sample we collected from the RA patient population. Moreover, this patient number exceeded the number of an earlier RA patient population, in which it displayed very high correlations between DAS28-MCP-1 and DAS28-ESR scores or and DAS28-CRP scores as reported^17^.

The SPSS 16·0 software package was used for all data analysis. Correlations of DAS28-MCP-1 with DAS28-ESR and DAS28-CRP scores were estimated by using Pearson correlation coefficients (r values) at each assessment. The correlation of DAS28-MCP-1 scores with SDAI scores was analysed by Spearman’s correlation coefficients (ρ values). Since some patients were missing at Month three, six, nine and twelve, missing patients’ age and disease duration (disease duration indicates the time period from the initial arthritic symptoms to the time of enrollment.) were compared with enrolled patients. CRP and ESR levels stratified additional correlation analysis. Moreover, sex (women vs. men), disease duration (aliquots of one year), and rheumatoid factor (RF-positive vs. RF-negative) were used to divide patients’ disease activity scores into different parts for examining correlation of different DAS28 formulae. Bland–Altman plots of DAS28-ESR, DAS28-CRP or SDAI and DAS28-MCP-1 values were used to evaluate the magnitude of differences between these scores at different time points. Sensitivity to change, including change in values calculated with the different DAS28 formulae/SDAI, change in HAQ-DI, and change in bone erosion, was also analysed. The 95% confidence interval (95% CI) of correlation coefficients was calculated by being based on the method of Fisher’s Z transformation as appeared^20^. Comparison between correlation coefficients was done as described^21^.

ROC curves for change in different DAS28/SDAI scores versus change in HAQ-DI and change in bony erosion (high vs. low) were used to evaluate validity, which is expressed as AUC. A *P* value of less than 0.05 (two-tailed) was considered to indicate statistical significance. PASS Power Analysis and Sample Size Software (NCSS LLC, Kaysville, UT 84307, USA) was used to assess the statistical power of our sample size at baseline for performing the analyses.

The performance of the different scores to detect remission was demonstrated to include sensitivity, specificity, PPV, NPV, Youden index, diagnostic effectiveness and diagnostic odds ratio, which were calculated based on the modified ARA remission definition^5^ and the 2011 ACR/EULAR remission definition^23^.

## Supplementary information

Supplementary information.

## Data Availability

The data that support the findings of this study are displayed in the article and in the supplementary information. Others are available from the corresponding author upon reasonable request.
