# Supplementary material for: A new laboratory surrogate (Monocyte Chemotactic Protein-1) for Disease Activity Score28: a favourable indicator for remission in rheumatoid arthritis
Source: Sci Rep. 2020 May 19;10:8238. doi: 10.1038/s41598-020-65127-5 (PMC7237461; doi:10.1038/s41598-020-65127-5)
Supplement: Supplementary file 1 — Supplementary information. [file 41598_2020_65127_MOESM1_ESM.pdf]

A new laboratory surrogate (Monocyte Chemotactic Protein-1) for Disease Activity Score28: a favourable indicator for remission in rheumatoid arthritis

Lieh-bang Liou, Yao-Fan Fang, Chih Feng Tan, Jenn-Haung Lai, Shr-shian Jang, Ping-Han Tsai, Ting-chih Yeh

	DAS28-ESR	DAS28-MCP-1	DAS28-CRP	SDAI	HAQ-DI [a]
DAS28-ESR	-	0.830	0.909 [b]	0.847	0.554
DAS28-MCP-1	0.830	-	0.899	0.931	0.600
DAS28-CRP	0.909 [b]	0.899	-	0.927	0.611
SDAI	0.847	0.931	0.927	-	0.640
HAQ-DI	0.554	0.600	0.611	0.640	-

eTable 1. Correlation among different kinds of DAS28 scores, SDAI scores, and HAQ-DI scores in 835 visits. DAS28-ESR: disease activity score 28 (DAS28) with inclusion of ESR; DAS28-MCP-1: DAS28 with inclusion of MCP-1; DAS28-CRP: DAS28 with inclusion of CRP; SDAI: simplified disease activity index; HAQ-DI: health assessment questionnaire-disability index. 835 visits included M0, M3, M6, M9 and M12 together. Shown above are *P*-values by Spearman's correlation except where indicated by Pearson correlation [b]. All *P*-values were less than 0.001.

[a] The 95% confidence interval for correlation with HAQ-DI (the far right column) is (0.505, 0.599), (0.555, 0.642), (0.567, 0.652) and (0.598, 0.678), respectively, from top to bottom, not statistically significant due to overlapping of 95% confidence intervals.

	Women (n = 142)	Men (n = 36)	RF-positive (n = 100)	RF-negative (n = 78)
Baseline	0.863 [a] (0.814-0.900)	0.796 [a] (0.633-0.891)	0.856 [b] (0.793-0.901)	0.835 [b] (0.752-0.892)
Month 6	0.877 (0.831-0.911)	0.778 (0.585, 0.888)	n.d.	n.d.
Month 12	0.851 (0.797-0.891)	0.778 (0.589-0.886)	0.863 (0.798-0.908)	0.778 (0.674-0.842)
Biologics	0.826 [c] (0.771-0.869)	0.730 [c] (0.572-0.827)	0.812 [d] (0.686, 0.891)	0.763 [d] (0.600, 0.865)

eTable 2. Correlations of DAS28-MCP-1 scores with DAS28 scores in stratified analysis. RF: rheumatoid factor; n.d.: not done. All numbers are correlation coefficients by Pearson correlation (all *P* < 0.001). Shown above are correlation coefficients plus (95% confidence interval: 95% CI). The latter was calculated by being based on the method of Fisher's Z transformation as appeared in page 100-104[20] and then compared by a method in ref. 21. [a] not statistically significant due to overlapping of 95% CI; those gender comparisons for Month 6 and Month 12: not significant. [b]95% CI: not statistically significant; those for Month 12 RF subgroup gave test statistic Z = 1.688 and *P* = 0.046 (ref. 20). [c] Biologics in 232 visits: women (n = 167 visits) and men (n = 65 visits), 95% CI: not statistically significant. [d] Biologics in 232 visits: RF-positive (n = 48 visits, containing Baseline and Month 12) and RF-negative (n = 43 visits), 95% CI: not statistically significant.

	Women (DAS28-ESR/DAS28-MCP-1/ DAS28-CRP/SDAI)	Men (DAS28-ESR/DAS28-MCP-1/DAS28-CRP/SDAI)
All visits	0.909/0.840/0.865/0.864 (n=672 visits)	- [a] (n=163 visits)
Baseline	0.896/0.836/0.845/0.932 (n=142)	- [a] (n=36)
Month 3	0.878/0.831/0.831/0.828 (n=131)	- [a] (n=32)
Month 6	- [a] (n=132)	- [a] (n=31)
Month 9	- [a] (n=130)	- [a] (n=32)
Month 12	- [b] (n=137)	- [a] (n=32)

eTable 3. Area under the curves (AUC) for different kinds of DAS28 scores/SDAI versus HAQ-DI in men and women. AUC was calculated by receiver-operating-characteristic analysis. Abbreviations are represented as in eTable 1. HAQ-DI was cut-off at 1.44 for its ability to differentiate radiographic progression damage from non-progression (ref. 28). Confidence interval (95% CI): All visits Women [0.829-0.989/0.715-0.966/0.735-0.995/0.736-0.992], Baseline Women [0.783-1.009/0.695-0.983/0.643-1.046/0.866-0.999], Month 3 Women [0.858-0.971/0.801-0.974/0.895-0.989/0.586-1.070]: all not significantly different in each category. [a] no positive data. [b] only one visit value positive, non-usable.

	Remission status	Odds Ratio	Non-remission status
Fulfillment of modified ARA remission			
DAS28-ESR (<2.6)	36.99% (64/173)	Reference	0.45% (3/662)
DAS28-ESR (<2.4)	43.36% (62/143)	Reference	0.72% (5/692)
DAS28-ESR (<2.2)	50.44% (57/113)	Reference	1.39% (10/722)
DAS28-ESR (<2.0)	53.49% (46/86)	Reference	2.80% (21/749)
DAS28-ESR (<1.8)	60.71% (34/56)	Reference	3.98% (31/779)
DAS28-MCP-1 (<2.6)	47.86% (67/140)	1.56 [a,b]	0.00% (0/695)
DAS28-MCP-1 (<2.4)	62.00% (62/100)	2.13 [a]	0.68% (5/735)
DAS28-MCP-1 (<2.2)	75.61% (62/82)	3.05 [a]	0.80% (6/753)
DAS28-MCP-1 (<2.0)	80.00% (48/60)	3.48 [a]	2.45% (19/775)
DAS28-MCP-1 (<1.8)	84.38% (27/32)	3.49 [a]	3.61% (29/803)
Fulfillment of 2011 ACR/EULAR remission definition			
DAS28-ESR (<2.6)	49.13% (85/173)	Reference	2.27% (15/662)
DAS28-ESR (<2.4)	47.55% (68/143)	Reference	4.62% (32/692)
DAS28-ESR (<2.2)	61.95% (70/113)	Reference	4.16% (30/722)
DAS28-ESR (<2.0)	67.44% (58/86)	Reference	5.61% (42/749)
DAS28-ESR (<1.8)	73.21% (41/56)	Reference	7.57% (59/779)
DAS28-MCP-1 (<2.6)	61.43% (86/140)	1.65 [a]	2.01% (14/695)
DAS28-MCP-1 (<2.4)	71.00% (71/100)	2.71 [a]	3.94% (29/735)
DAS28-MCP-1 (<2.2)	81.71% (67/82)	2.74 [a]	3.98% (30/753)
DAS28-MCP-1 (<2.0)	85.00% (51/60)	2.74 [a]	6.32% (49/775)
DAS28-MCP-1 (<1.8)	87.50% (28/32)	2.56 [a,c]	8.84% (71/803)

eTable 4. Comparison of remission rates by DAS28-ESR and DAS28-MCP-1 score-based statuses. The remission status of patients with rheumatoid arthritis was classified as in Tables 3 and 4. Abbreviations are the same as those in eTable 1 and Table 3. Inside parentheses are visit numbers.

[a] Odds ratios in each remission group represent individual DAS28-MCP-1 score-based remission statuses compared with DAS28-ESR remission statuses with the same cut point as the reference in each category of remission definition. All *P* were < 0.05, except [b] *P* = 0.053 and [c] *P* = 0.126.

	M3-M0	M6-M0	M9-M0	M12-M0
DAS28-ESR	0.423	0.504	0.497	0.470
DAS28-MCP-1	0.422	0.537	0.557	0.481
DAS28-CRP	0.432	0.493	0.528	0.466
SDAI	0.445	0.473	0.549	0.479
HAQ-DI	0.509	0.459	0.535	0.442

eTable 5. Area-under-the-curve for change in DAS28-ESR, DAS28-MCP-1, DAS28-CRP, SDAI and HAQ-DI scores versus change in bone erosion. Abbreviations were the same as in Table 1. Changes in bone erosion were calculated by subtracting Month 0 from Month 12 and the cut-off at 3 was used because 3 is bigger than the smallest detectable difference at 2.90 for bone-erosion readings as in Methods. M3-M0: DAS28, SDAI, and HAQ-DI scores taken at Month 3 minus those at Month 0; and similarly hereinafter. Five clinical scores in individual time periods are not different because of all having overlapping 95% confidence intervals (M6-M0: 0.405-0.604, 0.435-0.640, 0.392-0.593, 0.375-0.571, and 0.359-0.559, from top to bottom; M9-M0: 0.402-0.592, 0.462-0.652, 0.430-0.625, 0.448-0.650, and 0.440-0.631, from top to bottom).

Disease duration	DAS28-ESR	DAS28-MCP-1	DAS28-CRP	SDAI
≦ 1 yr (156 visits)	0.695	0.722	0.701	0.745
> 1 yr to ≦2 years (65 visits)	0.590	0.665	0.575	0.658
＞2 to ≦3 years (53 visits)	0.796	0.599	0.806	0.754
＞3 to ≦4 years (59 visits)	0.488	0.550	0.587	0.575
＞4 to ≦5 years (20 visits)	−0.122 [a]	0.745	0.519 [b]	0.629 [c]
＞ 5 years (482 visits)	0.453	0.510	0.527	0.552

eTable 6. Correlation of different kinds of DAS28 scores and SDAI scores with HAQ-DI scores for patient visits classified by different year-periods of disease duration. Abbreviations are designated as those in eTable 1. Visit numbers in parentheses were RA patient visits combined from Baseline and Month 3, 6, 9 and 12 (all together 835 visits). Correlations with HAQ-DI were analysed by Spearman correlation and all $P < 0.001$ except where indicated: [a] $P = 0.609$, [b] $P = 0.019$, [c] $P = 0.003$.

	Enrolled patients	Missing patients	*P*-values (95% confidence interval)
Age (year-old)			
At Month 3	52.9 ± 11.1 (n = 163)	53.0 ± 14.6 (n = 15)	0.974 (−8.366 to 8.108)
At Month 6	53.2 ± 11.0 (n = 163)	49.9 ± 15.0 (n = 15)	0.430 (−5.236 to 11.688)
At Month 9	53.2 ± 11.0 (n = 162)	49.5 ± 14.7 (n = 16)	0.399 (−4.261 to 11.693)
At Month 12	53.0 ± 11.3 (n = 169)	50.8 ± 14.2 (n = 9)	0.658 (−8.793 to 13.226)
Disease duration (months)			
At Month 3	97.3 ± 86.3 (n = 163)	124.5 ± 102.6 (n = 15)	0.334 (−85.248 to 30.733)
At Month 6	100.7 ± 88.8 (n = 163)	87.8 ± 77.8 (n = 15)	0.553 (−31.868 to 57.581)
At Month 9	102.9 ± 87.4 (n = 162)	66.0 ± 87.6 (n = 16)	0.125 (−11.308 to 85.085)
At Month 12	100.7 ± 88.3 (n = 169)	78.4 ± 79.6 (n = 9)	0.437 (−39.649 to 84.156)

eTable 7. Comparison of demographic data between enrolled and missing RA patients in different months. Age and disease duration are expressed as mean ± SD. *P*-values were done by t-test.
